# Towards understanding the role of Receptor Expression Enhancing Protein 5 (REEP5) in cardiac muscle and beyond

**DOI:** 10.15698/cst2020.06.223

**Published:** 2020-04-15

**Authors:** Shin-Haw Lee, Sina Hadipour-Lakmehsari, Anthony O. Gramolini

**Affiliations:** 1 Translational Biology and Engineering Program, Ted Rogers Centre for Heart Research, Toronto, Ontario, Canada M5G1M1.; 2 Department of Physiology, Faculty of Medicine, University of Toronto, Toronto, Ontario, Canada M5S1M8.

**Keywords:** SR organization, cardiac myocytes, cardiac ER stress, zebrafish, heart function, heart development

## Abstract

The sarco-endoplasmic reticulum (SR/ER) is the largest membrane-bound organelle in eukaryotic cells and plays important roles in essential cellular processes, and in development and progression of many cardiac diseases. However, many aspects of its structural organization remain largely unknown, particularly in cells with a highly differentiated SR/ER network. In a recently published study led by Lee *et al.* (Nat Commun 11(1):965), we reported a cardiac enriched SR/ER membrane protein REEP5 that is centrally involved in regulating SR/ER organization and cellular stress responses in cardiac myocytes. *In vitro* REEP5 depletion in mouse cardiac myocytes resulted in SR/ER membrane destabilization and luminal vacuolization along with decreased myocyte contractility and disrupted Ca^2+^ cycling. Further, *in vivo* CRISPR/Cas9-mediated REEP5 loss-of-function zebrafish mutants showed sensitized cardiac dysfunction to heart failure induction upon short-term verapamil treatment. Additionally, *in vivo* adeno-associated viral (AAV9)-induced REEP5 depletion in the mouse demonstrated cardiac dysfunction with dilated cardiac chambers, increased cardiac fibrosis, and reduced ejection fraction. These results demonstrate the critical role of REEP5 in SR/ER organization and function.

## DISCOVERY OF REEP5

Cardiac membrane proteins play an important role in regulating normal beat-to-beat function of the heart given their importance in coordinating spontaneous contractions of cardiomyocytes in the heart. In hopes of discovering novel molecular regulators of heart function and potential therapeutic targets for heart disease, an initiative to create a blueprint of all critical membrane and membrane-associated proteins in the heart was carried out in 2015 (Sharma *et al.*, Nat Commun 6:8391). Subsequent bioinformatic and rank-ordered analyses prioritizing evolutionarily conserved, cardiac-enriched, and poorly annotated cardiac membrane proteins identified numerous proteins, including REEP5.

## REEPS IS A CARDIAC-ENRICHED SR/ER MEMBRANE SHAPING PROTEIN

Our bioinformatic data across various human tissues and immunoblot analysis of multiple mouse organs suggested that although the expression of REEP5 was ubiquitously expressed across organs, it was found preferentially expressed in muscle cells with the most abundant expression in the cardiac ventricle. High-resolution three-dimensional co-immunofluorescence imaging of endogenous REEP5 in cardiac myocytes showed localization of REEP5 to different functional domains of the SR/ER network in the myocyte including the tubular/longitudinal SR/ER network where protein synthesis, trafficking, and modification takes place. REEP5 was also localized to the junctional SR, which is closely tethered to the cell surface and mainly responsible for Ca^2+^ cycling and the regulation of excitation-contraction coupling in the myocyte. These results led to the hypothesis that physiological expression of REEP5 is important for SR/ER function, and ultimately, contraction of the cardiac myocytes.

Depletion of REEP5 in cardiac myocytes resulted in SR/ER membrane destabilization and the formation of prominent luminal vacuoles. Morphologically, without REEP5, the SR membrane network failed to form high curvature SR/ER tubules that are required for proper association with t-tubules, and propagation of cardiac excitation-contraction coupling signals. Interestingly, REEP5 interactome analysis using mass spectrometry and co-immunoprecipitation studies elucidated REEP5 interactions with several members of the reticulon (RTN) and atlastin (ATL) families of proteins, as well as cytoskeleton associated protein 4 (CKAP4). These interacting proteins have been linked previously to ER membrane-shaping functions including high curvature formation, tubule fusion, and intraluminal spacing. Previous studies into the role of many of these proteins in the ER have mainly focused on yeast and *Xenopus laevis*. However, it is now apparent that members of these protein families, likely acting in a coordinated manner, will be important for specialized ER structure and function in unique mammalian cell populations and cardiac SR structure and biology as primary cardiac myocytes are fundamentally more complex. For instance, REEP5 is the dominant REEP protein in the heart among its other five members of the REEP family of proteins, named REEP1 to REEP6. Notably, REEP1, a neuro-enriched REEP protein, and its interaction with ATL1 has been shown to be important for maintaining ER tubular membrane in corticospinal neurons; defects in ER organization and this REEP1-ATL1 interaction have been shown to be the predominant pathogenic mechanism of hereditary spastic paraplegia, a neurodegenerative disease of the upper motor neurons. Our work in identifying REEP5-ATL3 interactions in cardiac myocytes would suggest that, similar to REEP1-ATL1 interactions in corticospinal neurons, normal cardiac SR/ER function and disruptions in REEP5 network interactions could be pathogenic and result in the development of cardiomyopathy. Similar insight would be anticipated given the specific cellular distribution and enrichment of REEPs, ATLs, and RTNs in a tissue- and cell-specific manner.

## REEP5 REGULATES CARDIAC CELL STRESS-INDUCED RESPONSES

**Figure 1:**
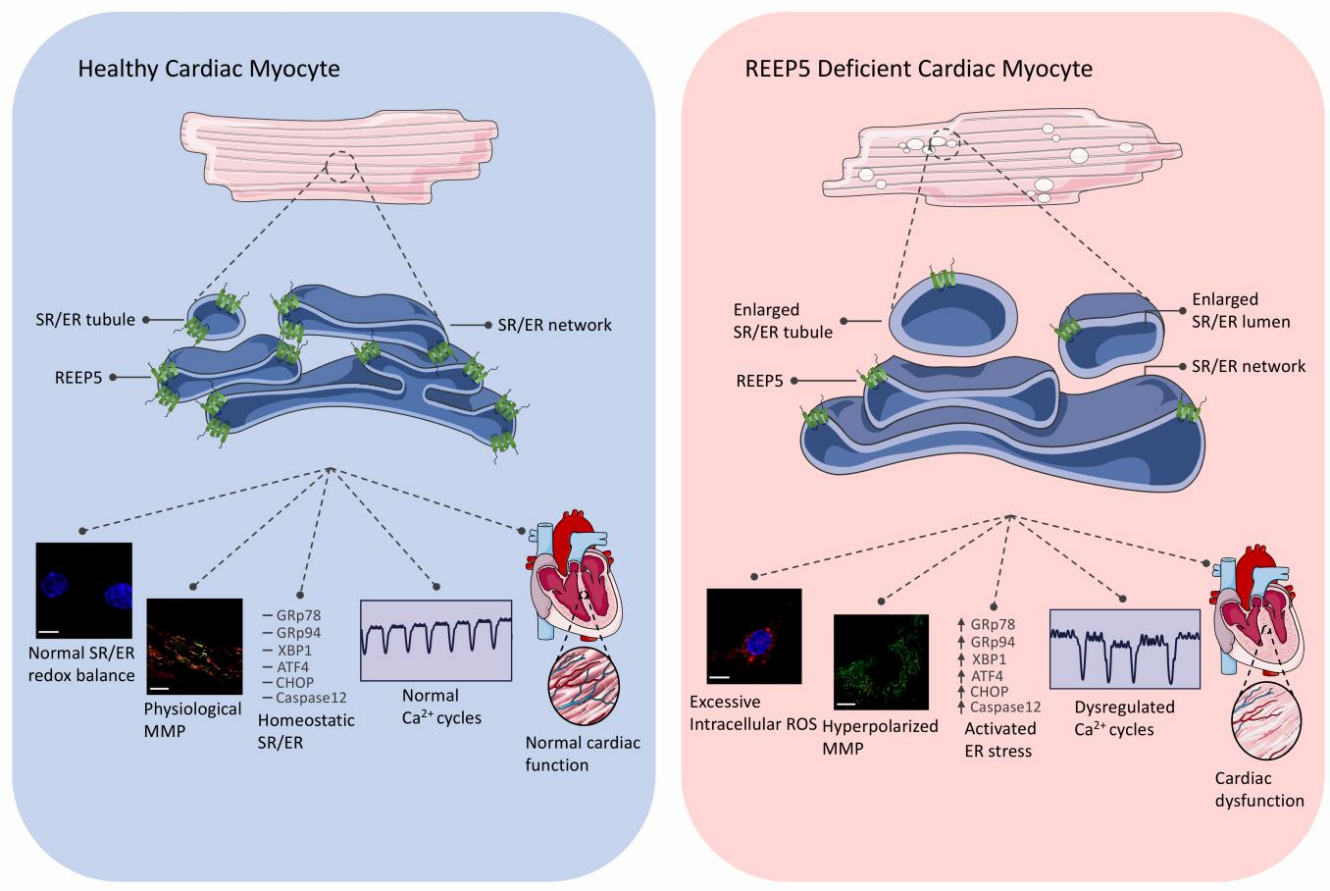
FIGURE 1: Summary of major functions of REEP5 in cardiac myocytes. REEP5 is a SR/ER membrane protein responsible for generating high membrane curvature to stabilize the highly differentiated SR network in cardiac myocytes. Normal expression of REEP5 under healthy physiological conditions (left) is required for proper formation, maintenance, and function of the SR/ER in the myocyte; loss of REEP5 (right) results in SR membrane vacuolization, excessive intracellular ROS, hyperpolarized mitochondrial membrane potential, activated ER stress pathways, dysregulated Ca^2+^ cycles, and cardiac dysfunction.

An anecdote saying “*structure leads to function*” applies well in the fields of cardiac biology and development. We demonstrated that REEP5 depletion-induced vacuolization of the SR/ER membrane correlates with elevated levels of intracellular reactive oxygen species (ROS), dissipated mitochondrial membrane potential, dysregulated Ca^2+^ cycles, and increased expression levels of ER stress markers and ER-dependent apoptotic marker, caspase 12 (**Figure 1**). Importantly, we showed that the C-terminus region of REEP5 in the cytosol is the link to REEP5-induced membrane vacuolization phenotype observed in cardiac myocytes; overexpression of the C-terminus truncated mutant of REEP5 led to severely vacuolated SR/ER. This data supports the existing evidence in the field that the conserved amphipathic helix domain in the C-terminus is essential for membrane stabilization support. These morphological and functional defects translated into lethal cardiac phenotypes which included degenerating muscle fibers and reduced heart rate, ejection fraction, and cardiac output in our preclinical zebrafish and mouse models of REEP5 depletion *in vivo*. Our study provided a detailed description of the fundamental role of REEP5 in SR/ER organization in the myocyte in the heart. However, future studies focused on understanding the precise molecular mechanism of action by which REEP5 may regulate ROS production and the ER stress pathways in the myocyte, for example, would prove invaluable insight into direct role of these proteins in SR/ER formation and maintenance. In fact, such studies may even help determine the therapeutic potential of REEP5 in heart disease as sustained ER stress has emerged as an important contributor to a wide range of prevalent human diseases including cardiomyopathies and congestive heart failure.

## NEW INSIGHTS INTO HEART FAILURE DEVELOPMENT AND PROGRESSION

Extensive investigations into heart disease have highlighted the SR/ER as a focal site for the initiation and progression of many heart diseases. From our depletion and knockout studies, it is evident that REEP5 is important in the normal functioning of the heart and potentially has a role in cardiac disease. The findings in *in vivo* REEP5-depleted mice suggest a dilated cardiomyopathy (DCM) phenotype linked with upregulation of cell stress markers. Heart failure is an increasingly common disease which has several different etiologies, some genetic and some acquired. Although over 100 genes have been associated with familial cardiomyopathy and heart failure, there are still many cases where the underlying cause of the disease remains unknown. From the initial data, it would appear likely that REEP5 may be linked to some of these idiopathic cases. Further genetic investigation of these cases would appear warranted, and may provide insight into understanding the mechanisms behind the disease.

